# Extrinsic Factors Underlying Food Valuation in the Human Brain

**DOI:** 10.3389/fnbeh.2020.00131

**Published:** 2020-07-24

**Authors:** Kosuke Motoki, Shinsuke Suzuki

**Affiliations:** ^1^Department of Food Management, School of Food, Agricultural and Environmental Sciences, Miyagi University, Sendai, Japan; ^2^Institute of Development, Aging and Cancer, Tohoku University, Sendai, Japan; ^3^Brain, Mind and Markets Laboratory, Department of Finance, Faculty of Business and Economics, The University of Melbourne, Parkville, VIC, Australia

**Keywords:** food, reward, value, preference, decision-making, consumer psychology, fMRI

## Abstract

Subjective values for food rewards guide our dietary choices. There is growing evidence that value signals are constructed in the brain by integrating multiple types of information about flavor, taste, and nutritional attributes of the foods. However, much less is known about the influence of food-extrinsic factors such as labels, brands, prices, and packaging designs. In this mini-review article, we outline recent findings in decision neuroscience, consumer psychology, and food science about the effect of extrinsic factors on food value computations in the human brain. To date, studies have demonstrated that, while the integrated value signal is encoded in the ventromedial prefrontal cortex, information on the extrinsic factors of the food is encoded in diverse brain regions previously implicated in a wide range of functions: cognitive control, memory, emotion and reward processing. We suggest that a comprehensive understanding of food valuation requires elucidation of the mechanisms behind integrating extrinsic factors in the brain to compute an overall subjective value signal.

## Introduction

**Figure 1:**
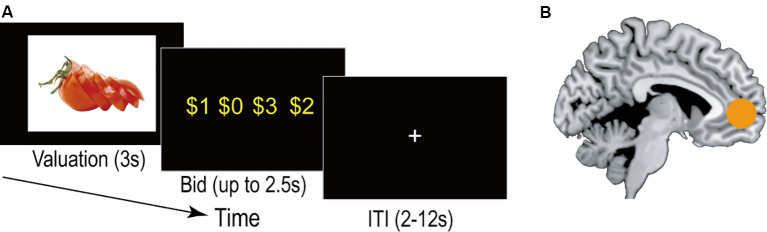
Value signals in the brain. **(A)** Example timeline of the experimental task (called Becker-DeGroot-Marschak auction: Becker et al., 1964). In this task, participants report their “willingness to pay” (i.e., subjective value) for food items. On each trial, they make a bid for one item in the context of *auction*. Importantly, the auction mechanism is carefully designed so that the optimal strategy for the participants is to always bid the number closest to their true subjective value for obtaining that item. The food image is adopted from Food-pics (Blechert et al., 2014). **(B)** Neural correlates of value signals. Subjective value signals are correlated with neural activity (blood-oxygen-level-dependent signal) in the vmPFC. vmPFC, ventromedial prefrontal cortex.

The valuation of food is central in our daily decision-making about what to eat. Dysfunctional food valuation is often associated with the development of obesity and eating-disorders (Yokum et al., 2011; Carnell et al., 2012; Foerde et al., 2015). Human neuroimaging studies have begun to uncover the neural basis of food valuation (Rangel, 2013; Giuliani et al., 2018) by combining functional magnetic resonance neuroimaging (fMRI) with careful assessment of subjective values for food items. In a typical experimental design, participants inside the MRI scanner are shown images of food and are asked to report their subjective values for each of those food items (see Figure 1A for details). Accumulating evidence suggests that the ventromedial prefrontal cortex (vmPFC) encodes subjective value signals for various types of potential outcomes including food rewards (Figure 1B; Chib et al., 2009; Lebreton et al., 2009; Bartra et al., 2013; Chikazoe et al., 2014; Clithero and Rangel, 2014; Gross et al., 2014).

How is it the value signal for a food reward is constructed in the human brain? Previous studies suggest that individuals compute the value of a food item by integrating information about multiple attributes from biologically relevant intrinsic factors (e.g., macronutrients, tastes, and flavors) to higher-order extrinsic factors (e.g., labels, brands, prices, and packaging designs; e.g., Steptoe et al., 1995; Satterthwaite and Fellows, 2018).

Researchers have examined the effects of nutrient factors with experimental designs using images of food as stimuli (Figure 1A; Tang et al., 2014; Suzuki et al., 2017; DiFeliceantonio et al., 2018). For example, food valuation is driven by the caloric content tracked in the vmPFC (Tang et al., 2014). Moreover, one study found that the subjective value of a food reward can be predicted by a linear combination of the constituent nutritive attributes (Suzuki et al., 2017). Multivariate decoding analyses on the neuroimaging data supported the possibility that information on the nutritive attributes of food is represented in the lateral orbitofrontal cortex (lOFC) and then integrated into the vmPFC to compute an overall subject value (Suzuki et al., 2017). Notably, an additional analysis in this study carefully ruled out the possibility that the lOFC contains information about low-level visual features of the food images (e.g., luminance and contrast). A subsequent study demonstrated supra-additive effects of fats and carbohydrates to food valuations beyond the linear combination (DiFeliceantonio et al., 2018), providing a potential account for overconsumption of high-fat/-carbohydrate food products (e.g., French fries).

Construction of the value signal through actual consumption/tasting (i.e., oral sensing) of the food has also been of considerable concern in human neuroscience. For instance, oral sensory representation of fat and sucrose have been found in the vmPFC, including the rostral anterior cingulate cortex (de Araujo and Rolls, 2004). Grabenhorst and colleagues further demonstrated that the vmPFC and lOFC track the pleasantness of oral fat texture *via* functional connectivity with the oral somatosensory cortex (Grabenhorst et al., 2010; Grabenhorst and Rolls, 2014).

Another line of study has attempted to characterize the neural encoding of quality, intensity, and preference for certain tastes and flavors (Small et al., 2007). One study demonstrated that pleasantness, or quality, of taste, is represented in the lOFC, while intensity is represented in the insular cortex and amygdala (Small et al., 2003). By combining careful experimental designs with multivariate analyses on neuroimaging data, recent studies have elaborated on how taste and flavor information is processed in the brain (e.g., Howard et al., 2015; Chikazoe et al., 2019; Avery et al., 2020). Chikazoe and colleagues showed taste qualities (sweet, salty, bitter, and sour) are represented in the insular cortex, which supports the notion that the insular is the primary gustatory cortex in humans (Small, 2010). Howard and colleagues revealed, by the careful manipulation of identities (qualities) of odor stimuli, that the lOFC encodes identity-specific values, while the vmPFC encodes subjective values independent of the identity. Although the extent to which taste and flavor contribute to the computation of subjective values beyond the nutritive and caloric contents remains elusive (de Araujo et al., 2020), these findings together suggest that the lOFC plays a pivotal role in representing multiple types of information about intrinsic factors of food (e.g., macronutrients, tastes, and flavors) which are then integrated in the vmPFC to compute an overall subjective value.

Despite the advancement in our understanding of the influences of intrinsic factors, much less is known about the effects of higher-order extrinsic factors on food valuation in the brain. Increasing evidence in consumer psychology and food science suggests that food valuation can be influenced by various factors outside of the food itself, such as labels, brands, prices, social information, and packaging designs (e.g., Okamoto and Dan, 2013; Higgs, 2015; Piqueras-Fiszman and Spence, 2015; Motoki et al., 2018, 2020). In this mini-review article, we discuss our current knowledge about how extrinsic factors affect food valuation in the brain while maintaining a focus on human neuroimaging (fMRI) studies. Although previous articles have discussed related issues (Plassmann et al., 2012; Okamoto and Dan, 2013; Stasi et al., 2018), the current review provides a new perspective by emphasizing how information about food-extrinsic factors gets integrated into the brain to compute an overall subjective value.

## Extrinsic Factors of Food Valuation

### Labels

In our everyday dietary choices, we often acquire a significant amount of information from the label attached to the food product (e.g., Piqueras-Fiszman and Spence, 2015; Motoki et al., 2020). A seminal study by de Araujo et al. (2005) examined how cognitive and semantic information modulates our food valuation. They exposed participants to an odor (isovaleric acid with cheddar cheese flavor) with different visual word labels, either “cheddar cheese” or “body odor.” The participants were found to rate the subjective value of the odor as more unpleasant when labeled “body odor” than when labeled “cheddar cheese.” Furthermore, the modulation of the unpleasantness rating was reflected in the neural activity in the vmPFC, suggesting that cognitive and semantic information can modulate food value signals in the vmPFC.

Enax et al. (2015a) compared two different ways of presenting the nutritional information in a food label. In the control condition, nutritional information was provided by a purely information-based textual label. Whereas, in the main condition, the same information was provided by a label with color codes: *green* and *red* signaling *healthy* and *unhealthy* foods, respectively. The behavioral data showed that the labeling method significantly affected participants’ willingness to pay (WTP) for food items. Nutritional labels with color codes increased WTP for healthy foods compared with the purely information-based labels. The neuroimaging data revealed that consistent with the previous findings (Plassmann et al., 2007), WTP for each food item was significantly correlated with neural activity in the vmPFC, regardless of the type of nutritional label. Furthermore, the red signals, indicating unhealthiness, were found to increase neural activity in the dorsolateral prefrontal cortex (dlPFC), which has been implicated in self-control (Hare et al., 2009), and modulate its functional connectivity with the vmPFC. These results are consistent with the notion that top-down self-control signals in the dlPFC modulate the food value signals represented in the vmPFC to enable an overall decision.

Top-down modulation of the brain valuation region is potentially more prominent in obese female participants, compared to the healthy controls (Ng et al., 2011). Ng et al. (2011) scanned obese and normal females with fMRI while the participants were anticipating receiving a milkshake. Critically, in the experiment, identical milkshakes were delivered with a label indicating it was either “regular” or “low-fat.” Relative to female participants with normal weight, obese participants showed greater activation in the vmPFC in response to receiving the regular labeled milkshake (vs. low-fat labeled one), which could contribute to excessive consumption of high-fat foods. A possible way to account for the result would be an excess top-down modulation of the food valuation in obese females, though the study did not provide neural evidence for the modulatory process (e.g., connectivity analyses).

Grabenhorst et al. (2008) examined the effects of labels including taste-related (“rich and delicious taste”) descriptions on food valuation by using umami-taste stimuli. They found that pleasantness ratings from tasting the stimuli were increased by the taste-related labels, while intensity ratings were not. Consistent with the behavioral results, the neural representation of the pleasantness in the vmPFC was enhanced by the taste-related labels, while the representation of the taste intensity in the insular cortex was intact. The results suggest that food value signals represented in the vmPFC are modulated by top-down information about taste. The same research group also investigated how the inclusion of health-related properties (e.g., “high in calories”), as well as taste-related properties (e.g., “sweet and juicy”) on labels, influenced food valuation and choice (Grabenhorst et al., 2013). The taste-related labels were found to enhance the neural representation of taste pleasantness in the amygdala, a core region in the emotional brain system (Pessoa, 2017). Similarly, the health-related labels enhanced activity in the amygdala, and the amygdala activity predicted the participant’s behavioral shift towards healthier choices, highlighting a potential role of emotion in food. An explanation of these findings (Grabenhorst et al., 2008, 2013) could be that top-down information about taste and healthiness in the amygdala modulates food value signals in the vmPFC. An interesting avenue for future research would be to elucidate the modulation process by employing connectivity analyses and brain stimulation techniques (see “Discussion” section for details).

In today’s society, many people are conscious about whether their food is produced ethically and sustainably. One study showed a positive effect of an “organic” label on food valuation (Linder et al., 2010). The authors found that participants’ WTP was significantly higher for food items possessing an organic label rather than those without. Furthermore, the presentation of organically labeled foods increased the neural activity in the ventral striatum, and the increased striatal activity accounted for the individual differences in concern for natural food gradients and daily organic food buying behavior. Another study examined the effect of “fair-trade” labels highlighting the ethical sustainability of the food product (Enax et al., 2015b). In the neuroimaging experiment, participants were asked to report their WTP for food items presented with or without a fair-trade label. The behavioral and neuroimaging data revealed that the presence of the label increased WTP, while also increasing neural activity in brain regions such as ventral striatum, anterior cingulate cortex, and superior frontal gyrus. The presentation of the label was also found to modulate functional connectivity between these regions and the vmPFC, which signaled WTP. These results suggest that the fair-trade label influenced the valuation of food in the vmPFC through the functional connectivity with the regions that track the label information.

### Price

Our daily purchasing behavior is guided not only by a preference for an item but also by the price (e.g., Jaeger, 2006). Underpriced goods are generally preferred, while overpriced goods are avoided. Hare et al. (2008) demonstrated in their neuroimaging experiment that both the WTP and the price of food determined participants’ purchasing decisions. Furthermore, the decision value of food (defined as the WTP minus the price) was found to be encoded in the lOFC, while WTP was encoded in the vmPFC. Another study (Knutson et al., 2007) suggests that, in purchasing various goods including food, the brain computes the decision value in the vmPFC, while the ventral striatum tracks WTP and the insular cortex tracks price.

In some cases, a high-priced item may be overvalued based on the belief that expensiveness implies enhanced quality. That is, the price information can reinforce the preference (i.e., WTP) for the item. For example, an expensive wine sometimes sells better than a comparable low-priced alternative. Plassmann et al. (2008) addressed this issue with a focus on the experienced pleasantness from consuming a glass a few drops of wine. In their neuroimaging experiment, participants were asked to sample different wines and report their experienced pleasantness. The critical manipulation was that, unknown to the participants, the identical wines were administered with different instructions about their retail price (i.e., a high price in half of the cases and a low price in the other cases). They found that price information about the wine was capable of manipulating participants’ experienced pleasantness. That is, the expectation of higher-priced wine increased subjective reports of flavor pleasantness. Moreover, the change in the pleasantness reports was reflected in the neural activity of the vmPFC. A follow-up study formally tested the tripartite relationship among price, pleasantness, and neural activity (Schmidt et al., 2017). The results of a multilevel mediation analysis revealed that the effect of price information on experienced pleasantness of wine tasting was mediated by neural activity in the brain valuation system including the vmPFC, ventral striatum, and anterior prefrontal cortex.

### Brand

Brand images can also influence our food choices. One study tested the effect of brand images by examining behavioral and neural responses to soft drink taste tests (McClure et al., 2004). In the experiment, the authors recruited participants who expressed either a preference for Coke or Pepsi. Within the experiment, participants were asked to choose between the two types of soda based on blind-tasting. The data analysis showed that participants’ choice pattern was not significantly correlated with their self-reported preference, implying that our daily food choice is predominated by brand images rather than experienced tastes. Furthermore, the preference that was estimated from the choice data, but not the self-report, was found to be represented in the vmPFC. Moreover, they showed that disclosure of the brand information, Coke but not Pepsi, increased participants’ preference for Coke and neural activity in dlPFC and hippocampus. A follow-up study (Koenigs and Tranel, 2008) examined the effect of the Coke brand cue in patients with lesions in the vmPFC. The authors demonstrated that the Coke label did not alter the patients’ preference, suggesting a causal role of vmPFC in processing brand information for food valuation.

Another study (Kühn and Gallinat, 2013) prepared an artificial beverage that consisted of equal parts of Coca Cola, Pepsi Cola, and River Cola. The artificial beverage was delivered to participants with different brand name cues: Coca Cola, Pepsi Cola, River Cola, and T Cola. The participants exhibited a preference for drinks associated with the famous brands (i.e., Coca and Pepsi Cola) over the others (i.e., River and T Cola) despite the chemical composition being identical. Furthermore, neural activity in the vmPFC was more responsive to the pleasantness rating from consuming the beverage when the cue signaled famous brands compared to the others. These two studies (McClure et al., 2004; Kühn and Gallinat, 2013) together suggest that brand images, possibly encoded in the dlPFC and the hippocampus, modulate value signals in the vmPFC.

### Social Information

Social information, such as the opinions of others, influences our judgment, preferences, and decision-making including food choice (e.g., Klucharev et al., 2009; Izuma, 2013; Higgs, 2015; Suzuki et al., 2016). Nook and Zaki (2015) investigated the social influence on food valuation. They conducted a neuroimaging experiment in which participants: (i) first rated how much they wanted to eat a series of foods; (ii) observed peer ratings for the foods; and (iii) again rated each of the food. As expected, the behavioral data showed a social conformity effect: that is, participants’ ratings about the foods were conformed to the peers’ ratings. At the neural level, an agreement between the participants’ and the peers’ ratings, as compared to disagreement, provoked neural activity in the ventral striatum, and the strength of the striatal activity predicted the individual differences in the degree of social conformity. Furthermore, the anterior prefrontal cortex was found to track information about the healthiness of the foods in the initial rating, but tracked popularity (i.e., information about peer ratings) in the second rating.

### Packaging Design

Packaging design can also affect our flavor expectation and preference for food (e.g., Basso et al., 2014; Spence et al., 2019; Tijssen et al., 2019). One study (Van der Laan et al., 2012) asked participants to choose between two options of the same snack contained within different packaging designs. The neuroimaging data showed that several brain regions, including the striatum, encoded outcomes of the package-based choices. Reimann et al. (2010) investigated how aesthetic packages and well-known brands influence our purchasing behavior. Comparing the two packages (i.e., aesthetic and standardized) and the two brands (i.e., well-known and unknown) conditions, they found that food items in aesthetics packages were more likely to be chosen despite higher prices when compared to well-known brands in standardized packages. The preference for aesthetic packages was reflected in the neural activity of the vmPFC, striatum, and middle to posterior cingulate cortex.

## Discussion

Consumer psychology and food science have a long history of demonstrating that our preference for a food reward is modulated by higher-order extrinsic factors (e.g., labels, brand images, prices, social information, and packaging designs; e.g., Okamoto and Dan, 2013; Higgs, 2015; Piqueras-Fiszman and Spence, 2015). In this mini-review article, we have discussed recent advancements in decision neuroscience in our understanding of how extrinsic factors affect food value computation in the brain.

**Figure 2:**
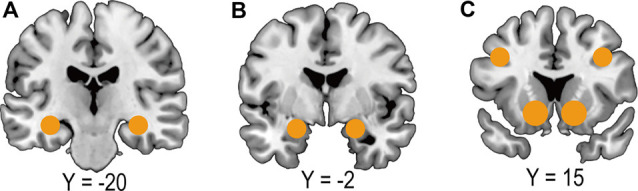
Brain regions encoding extrinsic factors of food.** (A)** Hippocampus. **(B)** Amygdala. **(C)** The dorsolateral prefrontal cortex (*top*) and ventral striatum (*bottom*).

Accumulating evidence from human neuroimaging studies has consistently demonstrated that, while vmPFC encodes overall value signals by integrating information about the extrinsic factors of foods (e.g., Enax et al., 2015a; Schmidt et al., 2017), the various extrinsic factors are encoded in diverse brain regions such as the dlPFC (McClure et al., 2004; Enax et al., 2015a), the amygdala (Grabenhorst et al., 2013), the ventral striatum (Linder et al., 2010; Van der Laan et al., 2012; Nook and Zaki, 2015; Schmidt et al., 2017), and the hippocampus (McClure et al., 2004; see Figure 2). These regions cover a wide range of brain functions: cognitive control (dlPFC), emotion (amygdala), reward processing (ventral striatum), and memory (hippocampus). Interestingly, the involvement of these diverse brain regions contrasts the food value computation based on intrinsic factors, in which information about macronutrients, tastes, and flavors appear to be integrated with the lOFC and sent to the vmPFC where the overall subjective value is computed (e.g., Suzuki et al., 2017).

The findings discussed in this review are broadly consistent with the notion that extrinsic factors of food reward modulate the value signal in the vmPFC through functional connectivity with multiple brain regions that track information about each extrinsic factor. However, more evidence is needed to deepen the understanding of how the multiple types of information become integrated within the brain to compute an overall food value (see Suzuki et al., 2015; Suzuki and O’Doherty, 2020 for similar issues in social decision-making). For example, to elucidate the integration process, it would be helpful to examine the nature of functional and anatomical connectivity among the brain regions engaged in the food valuation (e.g., vmPFC and dlPFC). Although several studies to date have aimed to address the issue by employing psychophysiological interaction analysis (Friston et al., 1997), the regression-based connectivity analysis cannot test for the directionality of the connections. Future studies could provide a more comprehensive view by combining various approaches that allow examination of the causal and anatomical interactions among brain regions, such as dynamic causal modeling of the neuroimaging data (e.g., Stephan et al., 2010; Hare et al., 2011), transcranial magnetic stimulation of the neural activity (e.g., Hill et al., 2017; Polanía et al., 2018), and diffusion tensor imaging (e.g., Assaf and Pasternak, 2008).

## Author Contributions

KM and SS wrote the manuscript.

## Conflict of Interest

The authors declare that the research was conducted in the absence of any commercial or financial relationships that could be construed as a potential conflict of interest.
